# Association of Physical Fitness Performance Tests and Anthropometric Indices in Taiwanese Adults

**DOI:** 10.3389/fphys.2020.583692

**Published:** 2020-11-20

**Authors:** Po-Hung Chen, Wei Chen, Cheng-Wei Wang, Hui-Fei Yang, Wan-Ting Huang, Hsiu-Chen Huang, Che-Yi Chou

**Affiliations:** ^1^Division of Occupational Therapy, Ditmanson Medical Foundation Chiayi Christian Hospital, Chiayi City, Taiwan; ^2^Department of Psychiatry, Ditmanson Medical Foundation Chiayi Christian Hospital, Chiayi City, Taiwan; ^3^Department of Community Health, Ditmanson Medical Foundation Chiayi Christian Hospital, Chiayi City, Taiwan; ^4^Clinical Medicine Research Center, Ditmanson Medical Foundation Chiayi Christian Hospital, Chiayi City, Taiwan; ^5^Department of Rehabilitation, Ditmanson Medical Foundation Chiayi Christian Hospital, Chiayi City, Taiwan; ^6^Division of Nephrology, Asia University Hospital, Wufeng, Taichung, Taiwan; ^7^Department of Post-baccalaureate Veterinary Medicine, Asia University, Wufeng, Taichung, Taiwan; ^8^Kidney Institute and Division of Nephrology, China Medical University Hospital, Taichung, Taiwan

**Keywords:** anthropometric indices, physical fitness performance, body mass index, waist-to-height ratio, 60-second sit-up, sit-and-reach, endurance, flexibility

## Abstract

**Background:**

The association between physical fitness performance tests and anthropometric indices is not clear. The study aims to explore the association between physical fitness performance and anthropometric indices in Taiwanese community-dwelling adults. This may help in monitoring anthropometric indices to improve physical fitness.

**Methods:**

We recruited 2216 participants aged 23–64 years between 2014 and 2017. Physical fitness performance, including abdominal muscular endurance (60-s sit-up test), flexibility (sit-and-reach test), and cardiorespiratory endurance (3-min step test), was evaluated in all participants. The association of the physical fitness performance and anthropometric indices, including body mass index (BMI), waist circumference (WC), waist-to-hip ratio (WHR), and waist-to-height ratio (WHtR), was analyzed using linear regression, with adjustments for age and gender.

**Results:**

Body mass index was negatively associated with abdominal muscular endurance (*p* < 0.001) and cardiorespiratory endurance (*p* < 0.001). Neither BMI, WC, WHR, nor WHtR were significantly associated with flexibility. Abdominal muscle endurance, flexibility, and cardiorespiratory endurance were significantly lower in obese participants when obesity was defined using a BMI of ≥27, 30, and 35 kg/m^2^. Participants with central obesity that was defined as WC ≥ 90 cm in men and 80 cm in women and WHtR ≥ 0.6 had lower abdominal muscular endurance than those without central obesity.

**Conclusion:**

Body mass index is associated with abdominal muscular endurance and cardiorespiratory endurance in a reverse J-shaped manner. None of the anthropometric indices are significantly associated with flexibility. Obesity defined by BMI is linked to worse physical fitness performance and obesity defined using WHtR is linked to lower abdominal muscular endurance in Taiwanese community-dwelling adults.

## Background

Higher levels of physical fitness are linked to better health in the elderly and decreased risk of chronic disease in later life (Pedrero-Chamizo et al., 2015). Lower levels of physical fitness are associated with chronic conditions (Stathokostas et al., 2015). Anthropometric indices can be used as a baseline for physical fitness and to measure fitness progression (Staub et al., 2018). Anthropometric indices, including body mass index (BMI), waist circumference (WC), waist-to-hip ratio (WHR), and waist-to-height ratio (WHtR), are commonly used in clinic (Chou et al., 2008). The best indices for physical fitness are not uniformly agreed upon. Body mass index is associated with physical fitness in the elderly (Chang et al., 2015), women (Mathisen et al., 2018), and children (Zaqout et al., 2016). Waist-to-height ratio may be better than BMI in accessing obesity-related decreased physical activity (Lee et al., 2016a). The association between WC, WHR, and WHtR in physical fitness in adults is not clear. Exploring the association between physical fitness performance tests and anthropometric indices may help to select the ideal anthropometric indices to monitor and subsequently improve physical fitness. Physical fitness performance tests, such as a 60-second sit-up test for abdominal muscle endurance (Gabbett et al., 2008), sit-and-reach test for flexibility (Mier, 2011), and 3-min step test for cardiorespiratory endurance (Bell et al., 2017; Hong et al., 2019), are commonly used. Their association with health has been established in multiple studies (Mendes et al., 2016; Chrismas et al., 2019; Chen et al., 2020). The ideal anthropometric indices may be different in different physical fitness performance tests. This study aims to explore the association between anthropometric indices, including BMI, WC, WHR, and WHtR, and three physical fitness performance tests in Taiwanese community-dwelling adults.

## Materials and Methods

Participant recruitment complied with the Declaration of Helsinki and was approved by the institutional review board of Ditmanson Medical Foundation Chia-Yi Christian Hospital (IRB NO 2020024). Informed consent was obtained from all participants. Inclusion criteria were as follows: aged between 23 and 64, no cardiovascular disease, no chest pain during exercise, no syncope or loss of consciousness, and able to sign informed consent. Exclusion criteria were as follows: chest pain during exercise, blood pressure higher than 160/100 mmHg, knee pain after exercise, syncope, loss of consciousness during exercise, and unable to give informed consent.

We approached 3052 individuals who visited a community center every week for social activities between 2014 and 2017. 265 individuals refused to join the study and 479 individuals were excluded due to either being diagnosed with cardiovascular disease or chest pain or from having blood pressure high than 160/100 mmHg, knee pain after exercise, or syncope. About 2308 individuals had physical fitness performance tests and 2216 of them completed the tests. Ninety-two individuals were unable to complete the tests. We divided all participants into five groups according to their age: Group 1 (23–30 years), Group 2 (31–40 years), Group 3 (41–50 years), Group 4 (51–60 years), and Group 5 (61–64 years) (Yu et al., 2020). All participants performed tests of physical fitness performance, including abdominal muscular endurance (60-s sit-up test), flexibility (sit-and-reach test), and cardiorespiratory endurance (3-min step test). Anthropometry indices, including height, weight, WC, and hip circumference, was measured at enrollment. Waist-to-hip ratio was calculated as WC divided by hip circumference. WHtR was calculated as WC divided by height.

We performed a 60-s sit-up test to measure abdominal muscular endurance. Participants were in a supine position with knees bent to 90°. The feet were flat on the floor and held by a partner. Arms were folded across the chest. For a successful sit-up to occur, the body was raised to a position of 90° to the ground (i.e., vertical) and then returned to the starting position. The sit-up action was to be continuous, with a single rest of no more than 2 s allowed between repetitions (Rikli and Jones, 2013). The number of sit-ups was recorded in *n*/min.

We applied a sit-and-reach test to assess low back flexibility. Participants sat on the test instrument and gradually reach forward as far as possible with knees extended. The test administrator checked to ensure the heel remained at the 35 cm mark and recorded the score in centimeters. The test was recorded twice, and the better score was used in the analysis (Rikli and Jones, 2013).

We used a 3-min step test to assess cardiorespiratory endurance. Participants performed 24 steps/min for 3 min on a 35 cm high step and were immediately seated after the step test. Their heart rates were counted for 30-s in intervals of 1.0–1.5 min, 2.0–2.5 min, and 3.0–3.5 min. Cardiorespiratory endurance was calculated as 180(s) × 100 divided by (the sum of the heart rate in the three 30-s recovery period) × 2 (Liu and Lin, 2007). All tests were performed on the same day with 30 min of rest between tests. To minimize the measurement error, all the tests were performed by the same team with the same protocol.

### Statistical Analysis

All descriptive statistics are presented as frequencies, percentages for categorical variables, and means ± standard deviations for continuous variables. The normality of the continuous data was tested using the Kolmogorov–Smirnov test. The association between anthropometric indices (BMI, WC, WHR, and WHtR) and physical fitness performance (60-s sit-up, sit-and-reach, and 3-min step tests) were visualized using the Lowess fit plot. The z-scores of BMI, WC, WHR, and WHtR were used to standardize the anthropometric indices to analyze them on a comparable scale. The association between anthropometric indices and physical fitness performance was further analyzed with linear regression with adjustments for age and gender, because age and gender are confounders of physical fitness performance. The regression coefficients and the 95% confidence intervals (CI) of the coefficients of BMI, WC, WHR, and WHtR were calculated using linear regression. Physical fitness performance scores according to different cut-offs of BMI, WC, WHR, and WHtR were analyzed with *t*-test to determine the application of the cut-offs. The cut-offs of BMI were 27 kg/m^2^ for mild obesity, 30 kg/m^2^ moderate obesity, and 35 kg/m^2^ severe obesity (Chu, 2005; Chang et al., 2017). Central obesity was defined by a WC ≥ 90 cm in males and 80 cm in females (Ma et al., 2013), a WHR of over 0.9 in males and 0.85 in females (Lin et al., 2004), and WHtR of over 0.6 (Lee et al., 2016b). All analysis was done using STATA (version 13, StataCorp, TX, United States). A *p* < 0.05 was considered as statistically significant.

## Results

We enrolled 648 men and 1568 women in this study (Table 1). The average scores of all participants were abdominal muscular endurance 20.9 ± 12.7 *n*/min, flexibility 25.1 ± 11.8 cm, and cardiorespiratory endurance 56.6 ± 19.1 s. The abdominal muscular endurance (26.5 ± 10.8 n/min) and cardiorespiratory endurance (58.7 ± 20.9 seconds) of men were better than that (18.6 ± 12.7 *n*/min and 55.7 ± 18.3 s) of women (*p* < 0.001 and *p* < 0.001, *t*-test). The flexibility (27.0 ± 11.5 cm) of women were better than that (20.5 ± 11.3 cm) of men (*p* < 0.001). About 26.4% of participants were aged 31–40, 27.8% were aged 41–50, and 23.1% were aged 51–60. The percentage of participants aged 23–30 and 41–50 were higher and the percentage of participants aged 31–40 and 51–60 were lower in women. Men had a higher height (170 ± 7 cm), weight (72 ± 12 kg), and BMI (25.3 ± 3.7 kg/m^2^) than women had (158 ± 6 cm, 57 ± 10 kg, and 23.5 ± 3.8 kg/m^2^). The WC, hip circumference, and WHR were not different between men and women. The WHtR of men (0.47 ± 0.06) was lower than that (0.51 ± 0.07) of women.

**TABLE 1:** Characteristics of all participants.

**Characteristics**	**All *n* = 2216**	**Male *n* = 648**	**Female *n* = 1568**	***p***
Abdominal muscular endurance (n/min)	20.9 ± 12.7	26.5 ± 10.8	18.6 ± 12.7	<0.001
Flexibility (cm)	25.1 ± 11.8	20.5 ± 11.3	27.0 ± 11.5	<0.001
Cardiorespiratory endurance (s)	56.6 ± 19.1	58.7 ± 20.9	55.7 ± 18.3	<0.001
Age *n* (%)				
23–30	373 (16.8)	127 (19.6)	246 (15.7)	0.004*
31–40	584 (26.4)	187 (28.9)	397 (25.3)	
41–50	617 (27.8)	161 (24.8)	456 (29.1)	
51–60	511 (23.1)	128 (19.8)	383 (24.4)	
61–64	131 (5.9)	45 (6.9)	86 (5.5)	
Height (cm)	161 ± 8	170 ± 7	158 ± 6	<0.001
Weight (kg)	62.8 ± 124	72 ± 12	57 ± 10	<0.001
BMI (kg/m^2^)	24.0 ± 3.9	25.3 ± 3.7	23.5 ± 3.8	<0.001
Waist circumference (WC, cm)	80 ± 10	80 ± 10	80 ± 10	0.622
Hip circumference (cm)	96 ± 7	96 ± 7	97 ± 7	0.104
Waist-hip ratio (WHR)	0.83 ± 0.07	0.83 ± 0.07	0.83 ± 0.07	0.791
Waist-height ratio (WHtR)	0.50 ± 0.07	0.47 ± 0.06	0.51 ± 0.07	<0.001

*Chi-square test. BMI, body mass index.

**FIGURE 1:**
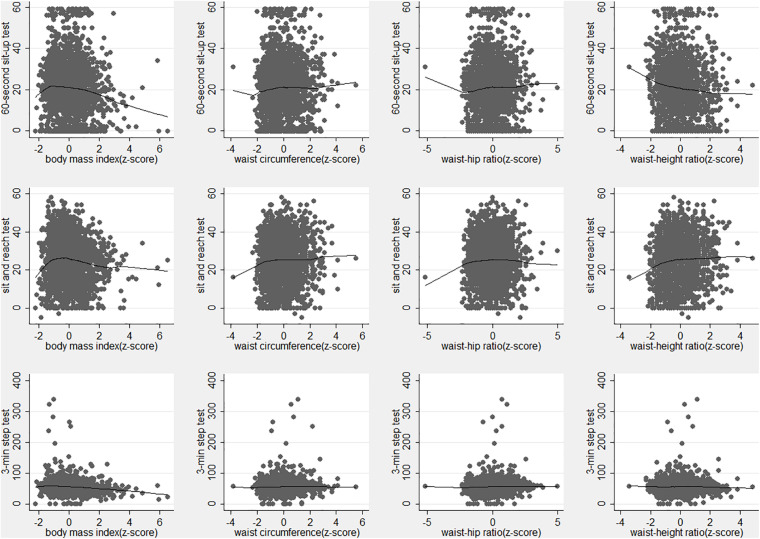
Lowess fit plot of 60-s sit-up (top row), sit-and-reach test (middle row), and 3-min step test (bottom row) vs body mass index (left column), waist circumference (middle left column), waist-hip ratio (middle right column), and waist-height ratio (right column) in all participants.

We visualized the association of physical fitness performance against anthropometric indices according to the z-score of BMI, WC, WHR, and WHtR using the Lowess fit plot (Figure 1). The abdominal muscular endurance (Figure 1 top row) and cardiorespiratory endurance (Figure 1 third row) were decreased with the increase of BMI, especially at a higher BMI. The fit lines of BMI, WC, WHR, and WHtR were flat in the plots for flexibility (Figure 1 second row). The fit lines of WC, WHR, and WHtR were flat in the plots of abdominal muscular endurance and cardiorespiratory endurance.

Body mass index was negatively associated with abdominal muscular endurance and cardiorespiratory endurance (*p* < 0.001 and *p* < 0.001). The regression coefficient was −1.40 (95% CI: −1.88 and −0.93) and −2.99 (95% CI: −3.81 and −2.17) in linear regression with adjustments for age and gender (Table 2). Waist circumference, WHR, and WHtR were not significantly associated with abdominal muscular endurance, flexibility, and cardiorespiratory endurance.

**TABLE 2:** Regression coefficients and their confidence intervals of anthropometric indices in association with physical fitness performance in linear regression with adjustments for age and gender.

**Anthropometric indices (z-score)**	**Abdominal muscular endurance**			**Flexibility**			**Cardiorespiratory endurance**		
	**Coef**	**95%**	**CI**	**Coef**	**95%**	**CI**	**Coef**	**95%**	**CI**
BMI	−1.40*	–1.88	–0.93	–0.30	–0.79	0.20	−2.99*	–3.81	–2.17
WC	0.25	–0.20	0.70	–0.19	–2.02	1.65	1.76	–1.28	4.80
WHR	–0.21	–1.02	0.59	0.06	–0.77	0.90	0.58	–0.80	1.96
WHtR	–0.15	–0.60	0.31	0.31	–1.54	2.17	–1.88	–4.94	1.18

Coef: coefficients, BMI, body mass index; WC, waist circumference; WHR, waist-hip ratio; WHtR, waist-height ratio. *p < 0.01.

The physical fitness performance according to different cut-offs of BMI, WC, WHR, and WHtR of obesity are shown in Table 3. Participants with a BMI ≥27 kg/m^2^ (mild obesity), ≥30 kg/m^2^ (moderate obesity), and ≥35 kg/m^2^ (severe obesity) had significantly lower abdominal muscular endurance, flexibility, and cardiorespiratory endurance than those without (*p* < 0.001, *p* < 0.001, and *p* < 0.001, *t*-test). Participants with a WC ≥ 90 cm in men and 80 cm in women had lower levels of abdominal muscular endurance than those without (*p* = 0.01). The flexibility and cardiorespiratory endurance were not different in the cut-off of WC. The physical fitness performance was not different in participants with a WHR ≥ 0.9 in men, ≥0.85 in women, and those without. Participants with a WHtR ≥ 0.6 had a lower level of abdominal muscular endurance than those without (*p* = 0.02).

**TABLE 3:** Mean and standard deviations of physical fitness according to a different cut-off of anthropometric indices.

**Anthropometric indices**	**Abdominal muscular endurance (n/min)**	**Flexibility (cm)**	**Cardiorespiratory endurance (n/min)**
BMI (kg/m^2^)			
<27	21.4 ± 12.5	25.8 ± 11.9	57.6 ± 19.8
≥27, *n* = 431	18.8 ± 13.4*	22.1 ± 10.9*	52.3 ± 15.1*
<30	21.3 ± 12.7	25.3 ± 11.9	57.1 ± 19.2
≥30, *n* = 161	15.6 ± 11.7*	22.3 ± 9.9*	49.4 ± 16.1*
<35	21.0 ± 12.6	25.1 ± 11.8	56.7 ± 19.1
≥35, *n* = 23	12.5 ± 13.1*	20.6 ± 10.1*	40.6 ± 14.0*
WC (cm)			
None	21.5 ± 12.6	24.4 ± 12.0	56.5 ± 17.9
≥90 male, ≥80 female, *n* = 877	20.1 ± 12.8*	26.2 ± 25.5	56.7 ± 20.8
WHR			
None	20.9 ± 12.8	25.1 ± 11.9	56.4 ± 18.8
≥0.9 male, ≥0.85 female, *n* = 336	20.9 ± 12.2	25.2 ± 11.5	57.6 ± 21.0
WHtR			
<0.6	21.1 ± 12.6	25.0 ± 11.8	56.7 ± 19.4
≥0.6, *n* = 176	18.7 ± 13.0*	26.5 ± 12.0	55.4 ± 15.3

BMI, body mass index; WC, waist circumference; WHR, waist-hip ratio; WHtR, waist-height ratio. *p < 0.05 in *t*-test.

## Discussion

Body mass index was negatively associated with abdominal muscular endurance, cardiorespiratory endurance, and cardiorespiratory endurance in community-dwelling adults. Neither BMI, WC, WHR, nor WHtR were associated with flexibility. All three physical fitness performances decreased with the severity of obesity defined by BMI (*p* trend <0.001, linear regression), while obesity was defined as 27 kg/m^2^ for mild obesity, 30 kg/m^2^ for moderate obesity, and 35 kg/m^2^ for severe obesity using BMI (Chu, 2005; Chang et al., 2017). An optimal BMI (19–24 kg/m^2^) is associated with a better physical fitness performance and a BMI of <19 or BMI >24 is associated with decreased physical fitness performance. The reverse J curve association between BMI and physical fitness is supported by previous studies (Hemmingsson and Ekelund, 2005; Bradbury et al., 2017; Eddolls et al., 2018). Body mass index may be more correlated with lean body mass than WC-associated anthropometric indices and is linked to better abdominal muscular endurance cardiorespiratory endurance (El Ghoch et al., 2018).

Waist circumference, waist-hip ratio, and waist-height ratio are central obesity indices and may be correlated with flexibility (Chang et al., 2015). Central obesity is linked to metabolic syndrome, diabetes, hypertension, and cardiovascular disease. We did not find an association between anthropometric indices and flexibility because flexibility may be affected by back pain in the participants (Sadler et al., 2017). Elderly participants with a slightly higher waist circumference may have more muscle mass and therefore have a better physical fitness performance (Moreira et al., 2016; Mikkola et al., 2018). More studies are needed to explore types of exercise that may improve BMI or WC and to determine if the improvements in BMI or WC can lead to improvements in physical fitness performance and successful aging.

Limitations of this study include the cross-sectional study design, as we are not able to explore if keeping BMI to an optimal range can improve physical fitness performance. Second, the chance of unreported chronic diseases, such as diabetes, hypertension, or dyslipidemia, cannot be completely excluded. Third, there are more female participants in this study. Females are more likely to participate in community activities in Taiwan and selection bias cannot be excluded.

## Conclusion

Waist circumference-based anthropometric indices may be useful in monitoring decreased physical activity because of central obesity. Body mass index remains a useful anthropometric index in assessing physical fitness in adults because BMI is linked to abdominal muscular endurance and cardiorespiratory endurance in a reverse J-shaped relationship. Being underweight and having obesity defined by BMI are associated with decreased physical fitness in Taiwanese community-dwelling adults. This association was not observed in WC-based anthropometric indices. None of the anthropometric indices, including BMI, WC, WHR, and WHtR, are significantly associated with flexibility. More studies are needed to explore better anthropometric indices for flexibility.

## Data Availability Statement

The raw data supporting the conclusions of this article will be made available by the authors, without undue reservation.

## Ethics Statement

The studies involving human participants were reviewed and approved by institutional review board of Ditmanson Medical Foundation Chia-Yi Christian Hospital. The patients/participants provided their written informed consent to participate in this study.

## Author Contributions

P-HC and WC analyzed and interpreted the data. C-WW and H-FY made substantial contributions to the conception of the study. C-YC drafted the work. W-TH and H-CH substantively revised the work. All authors read and approved the final manuscript.

## Conflict of Interest

The authors declare that the research was conducted in the absence of any commercial or financial relationships that could be construed as a potential conflict of interest.
